# Supplementary figures and images for: Tamoxifen resistance alters sensitivity to 5-fluorouracil in a subset of estrogen receptor-positive breast cancer
Source: PLoS One. 2021 Jun 8;16(6):e0252822. doi: 10.1371/journal.pone.0252822 (PMC8186817; doi:10.1371/journal.pone.0252822)

S1 Fig

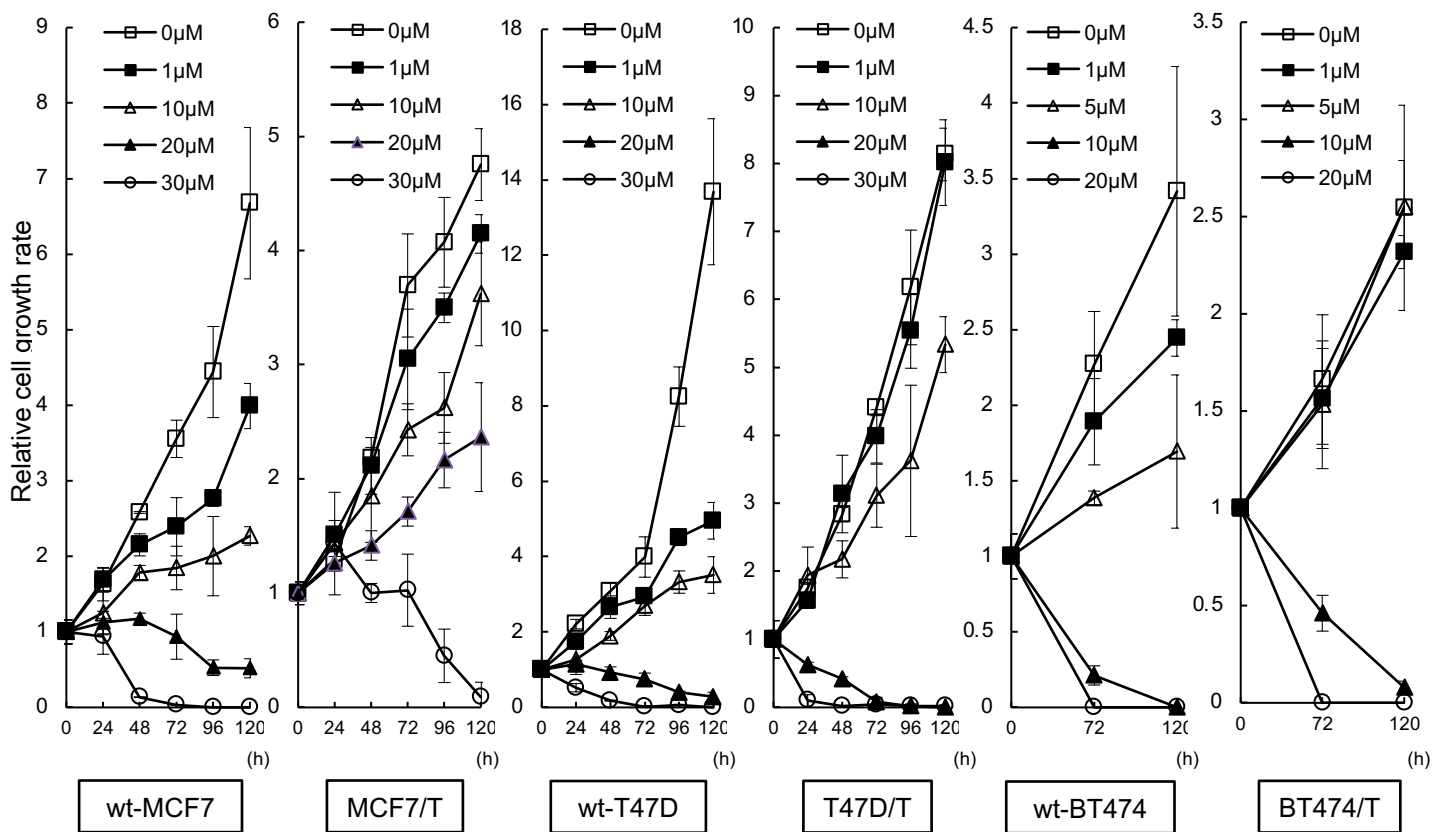

Supplement: S1 Fig — The growth inhibitory effects of TAM in wt-MCF7, MCF7/TAM, wt-T47D, T47D/T, BT474, and BT474/T was evaluated by cell proliferation assay. (A) The growth of wild-type and TAM-resistant MCF7, T47D, and BT474 cells treated with tamoxifen was measured by direct cell count. The relative proliferation rate was plotted by comparing the number of cells at each time point with the number at 0 h. The error bars represent the standard deviations of the values obtained from triplicate experiments. (PDF) [file pone.0252822.s001.pdf]

S2 Fig

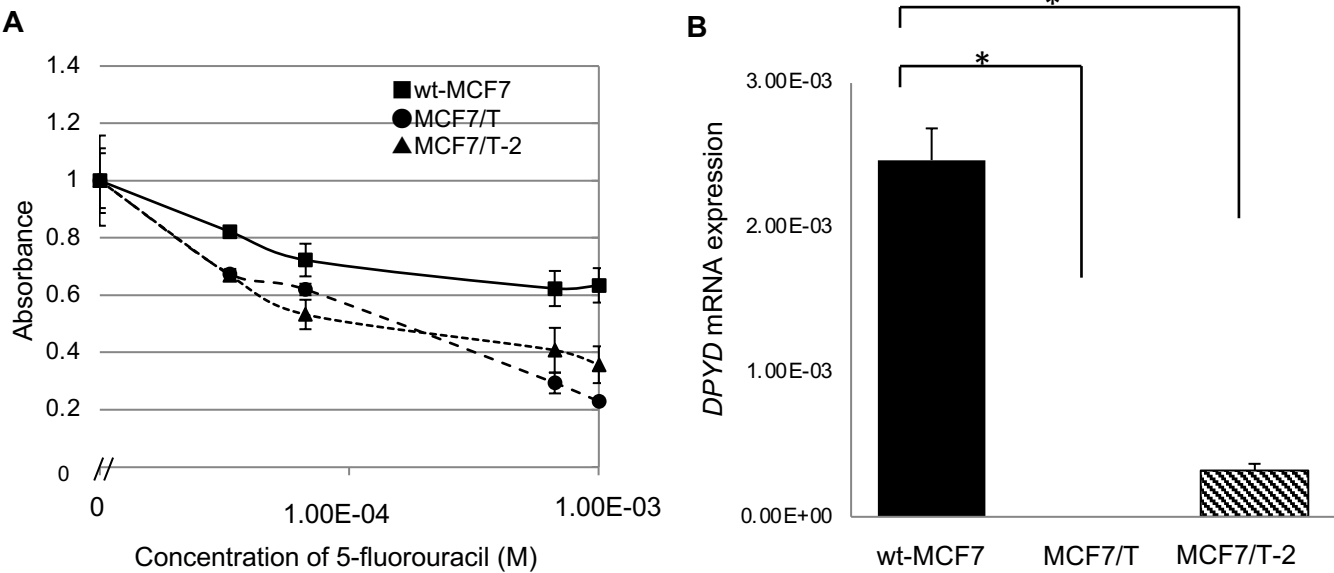

Supplement: S2 Fig — We had established several TAM-resistant sublines for MCF7 cells, and we tested 5-fluorouracil sensitivity by WST assay (A), and DPYD mRNA expression by real-time RT-PCR (B) in a representative clone, MCF7/T-2. MCF7-T2 demonstrated an increased sensitivity to 5-fluorouracil equivalent to MCF7/T, and showed a decreased expression of DPYD mRNA compared to wt-MCF7 cells. (PDF) [file pone.0252822.s002.pdf]

S3 Fig

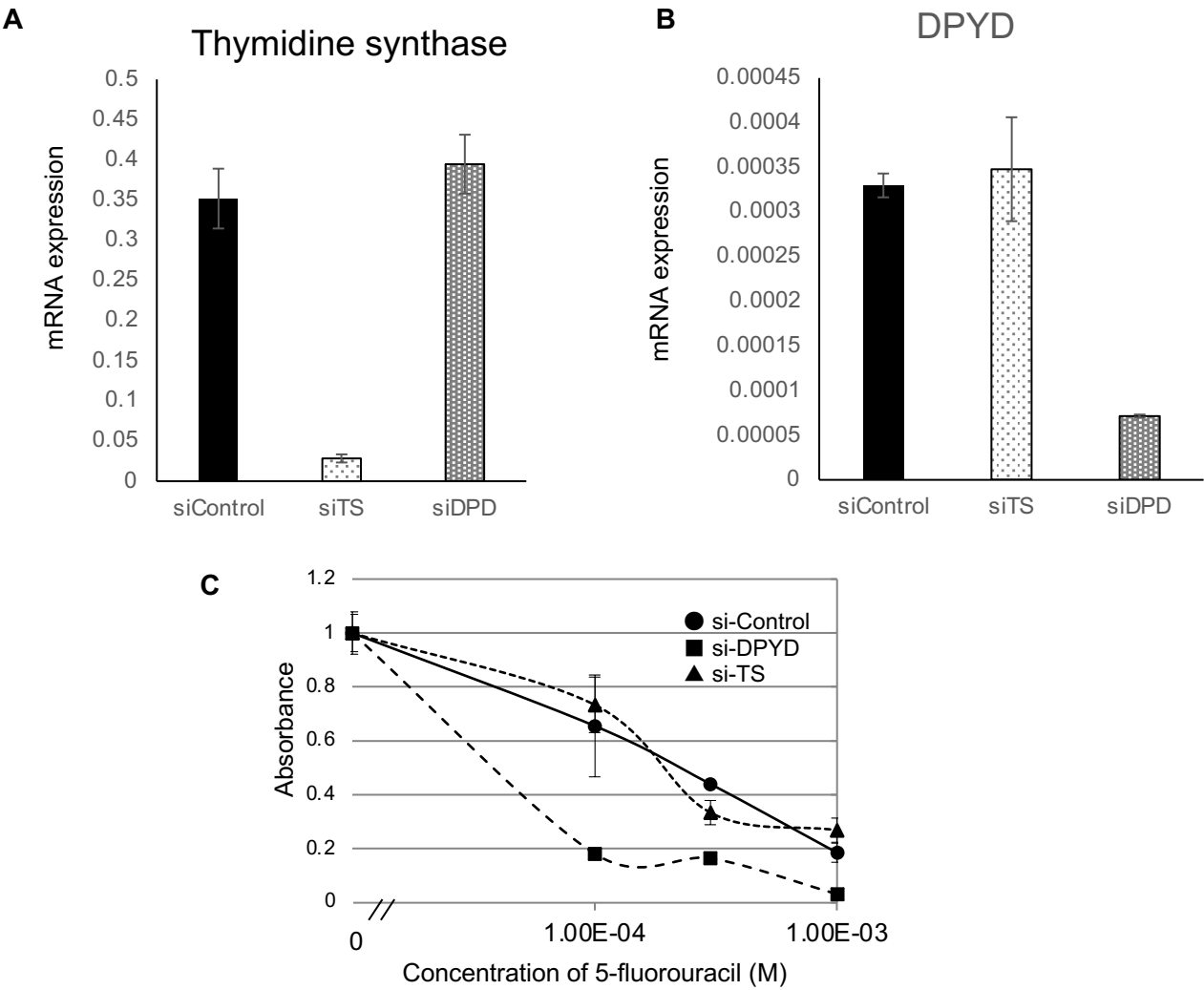

Supplement: S3 Fig — To evaluate whether thymidine synthase (TS) or dihydropyrimidine dehydrogenase (DPYD) were involved in sensitivity to 5-fluorouracil, we tested whether the knockdown of either enzyme would alter 5-fluorouracil sensitivity in wt-MCF7 cells. Inhibition of TS and DPYD mRNA expression was confirmed by real-time RT-PCR (A, B). The sensitivity to 5-fluorouracil was tested by WST assay (C). siRNA targeting of DPYD sensitized the wt-MCF7 cells to 5-fluorouracil, while siRNA targeting of TS did not alter the sensitivity to 5-fluorouracil. (PDF) [file pone.0252822.s003.pdf]

S4 Fig

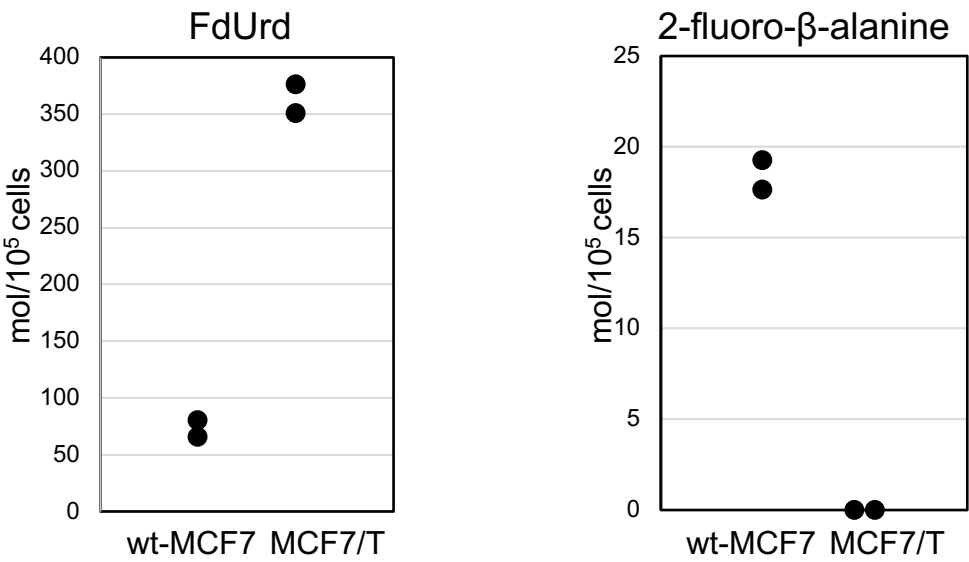

Supplement: S4 Fig — The intracellular concentrations of 5-fluorouracil metabolites, fluorodeoxyuridine (FdUrd, left panel) and 2-fluoro-β-alanine (FBAL, right panel) were quantitated by matrix-assisted laser desorption/ionization time-of-flight mass spectrometry (MALDI-TOF MS) as described in the S1 File. The amount of 5-fluorouracil active metabolite, FdUrd and FBAL were higher and lower in MCF7-T cells compared with those in wt-MCF7 cells, respectively. The experiment was done in duplicate. (PDF) [file pone.0252822.s004.pdf]
