# Supplementary material for: Tamoxifen resistance alters sensitivity to 5-fluorouracil in a subset of estrogen receptor-positive breast cancer
Source: PLoS One. 2021 Jun 8;16(6):e0252822. doi: 10.1371/journal.pone.0252822 (PMC8186817; doi:10.1371/journal.pone.0252822)
Supplement: S1 File — (DOCX) [file pone.0252822.s005.docx]

**Supplementary materials and methods**

**Small interfering RNA (siRNA) transfection**

The siRNAs for *TYMS* (L-004717) and the negative control (D-001810) were purchased from GE Healthcare (Buckinghamshire, UK). The siRNA for *DPYD* (#105956) was purchased from Invitrogen (Carlsbad, CA, USA). Transfection of each siRNA (10 nM) was performed using Lipofectamine RNAi-MAX (Thermo Fisher Scientific, Waltham, MA, USA) following the manufacturer’s instructions. At 48 h after transfection, the total RNA was extracted and 3 × 10^3^ cells/well were cultured in 96-well tissue culture plates and incubated for 72 h after adding various concentrations of 5-fluorouracil. Finally, the absorbance was measured using the WST solution.

**Extraction of metabolites of 5-fluorouracil**

MCF-7 and MCF-7/T cells were seeded in 60 mm^2^ dishes and incubated overnight. The dishes were then treated with 50 μM 5-fluorouracil and incubated for 72 h. The cells were washed with PBS to remove 5-fluorouracil and its extracellular metabolite. Cells were collected by trypsinization and centrifugation at 5000 rcf for 3 min. Each cell pellet was resuspended in 500 μL of 100% methanol and was frozen with liquid nitrogen for 10 min. Then, the pellet was thawed at room temperature with occasional vortexing, followed by sonication for 10 min. The samples were centrifuged at 15,000 rpm for 10 min at 4 ℃ and the supernatants were collected.

**Matrix-assisted laser desorption/ionization time-of-flight mass spectrometry (MALDI-TOF MS) for 5-fluorouracil metabolites**

Stock solutions (1 mM) of fluorodeoxyuridine (FdUrd) and 2-fluoro-β-alanine (FBAL) were prepared in methanol. Final concentrations were 25, 50, 100, 150, 300 μM for FdUrd and FBAL. The sample (0.5 μl) was added onto 0.5 μl CHCA (α-cyano-4-hydroxycinnamic acid) matrix and allowed to dry at room temperature. Mass spectra were examined with AB SCIEX TOF/TOF 5800 operated in positive ion reflectron mode. The calibration curves were analyzed as plots of peak area ratio of analyte/internal standard (CHCA) versus the analyte concentration. Then, raw data points were fitted to a linear regression and the determination coefficient (r^2^) of the linear regression was at least equal to 0.97 for compounds.
