# Supplementary material for: Tamoxifen resistance alters sensitivity to 5-fluorouracil in a subset of estrogen receptor-positive breast cancer
Source: PLoS One. 2021 Jun 8;16(6):e0252822. doi: 10.1371/journal.pone.0252822 (PMC8186817; doi:10.1371/journal.pone.0252822)

Uncropped image of Figure 1B

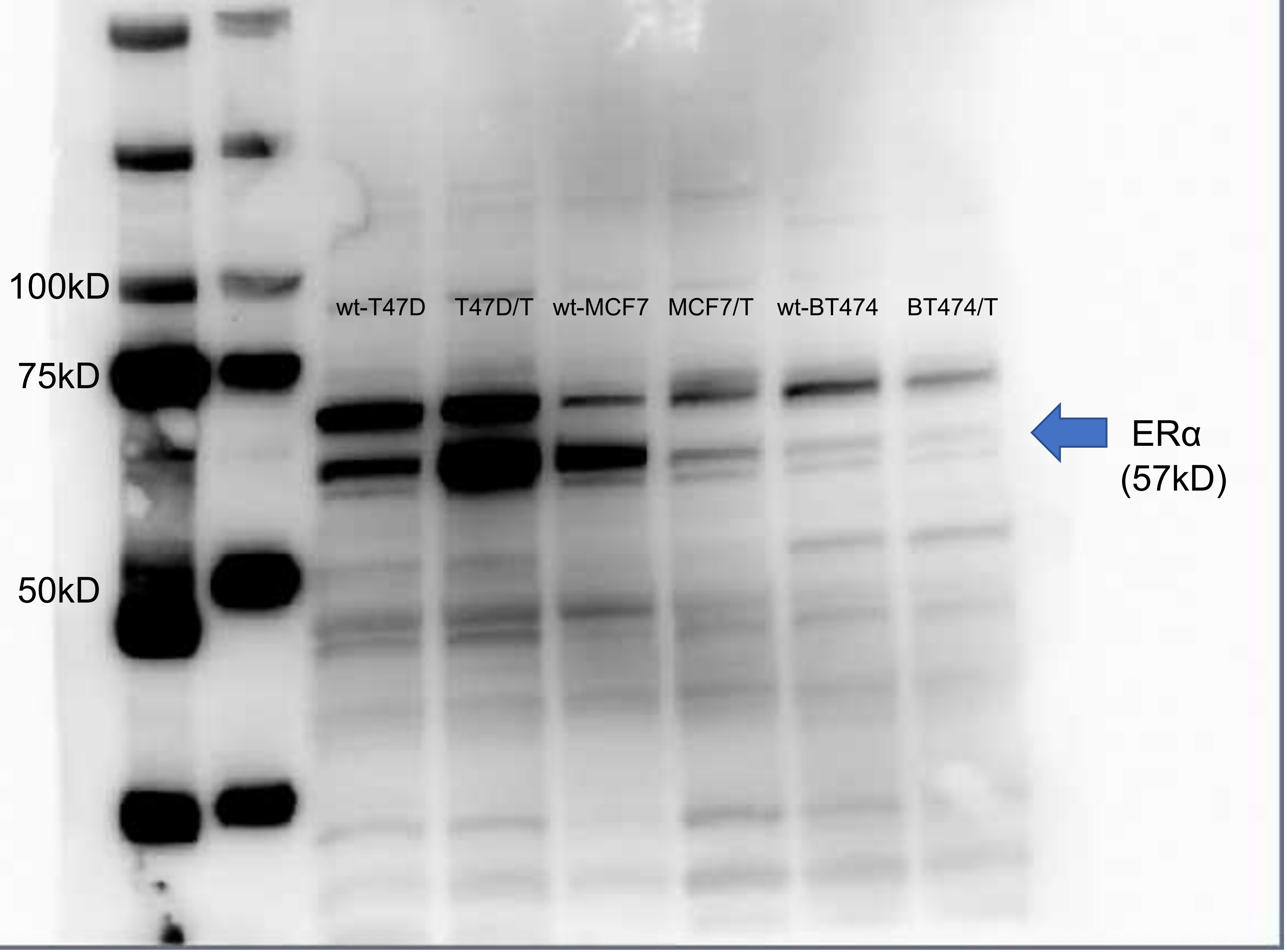

Uncropped image of Figure 1B

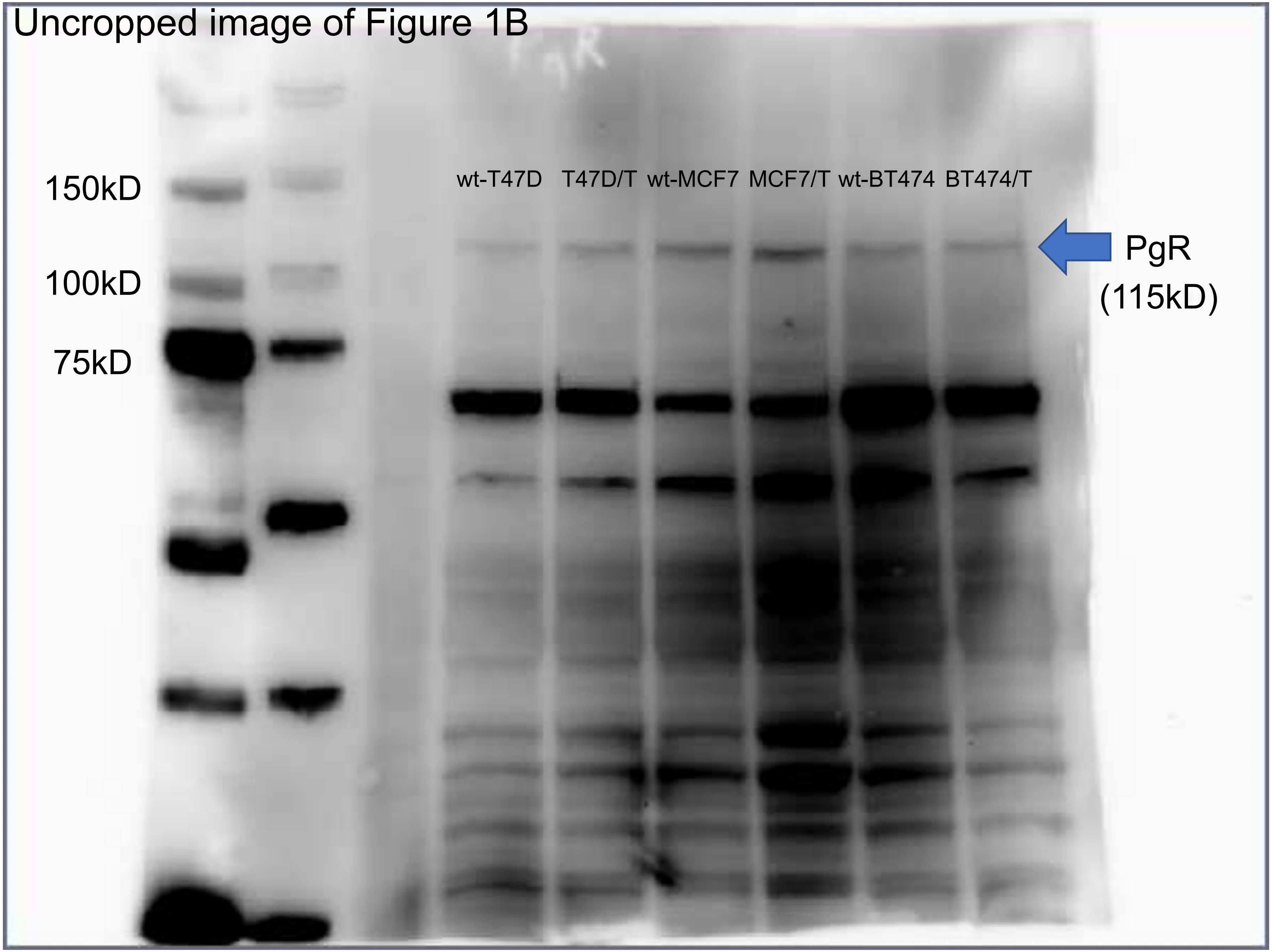

Uncropped image of Figure 1B

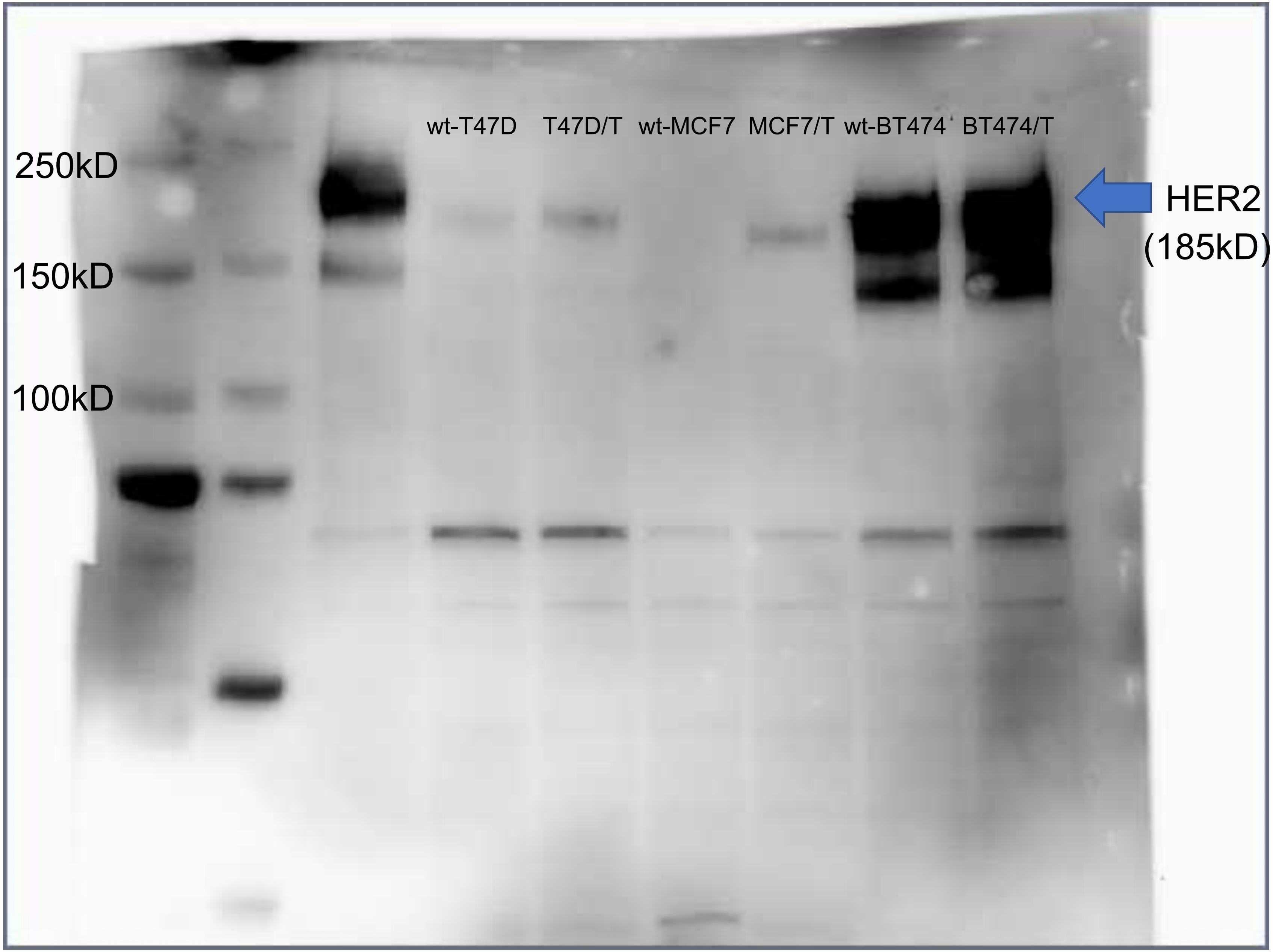

Uncropped image of Figure 1B

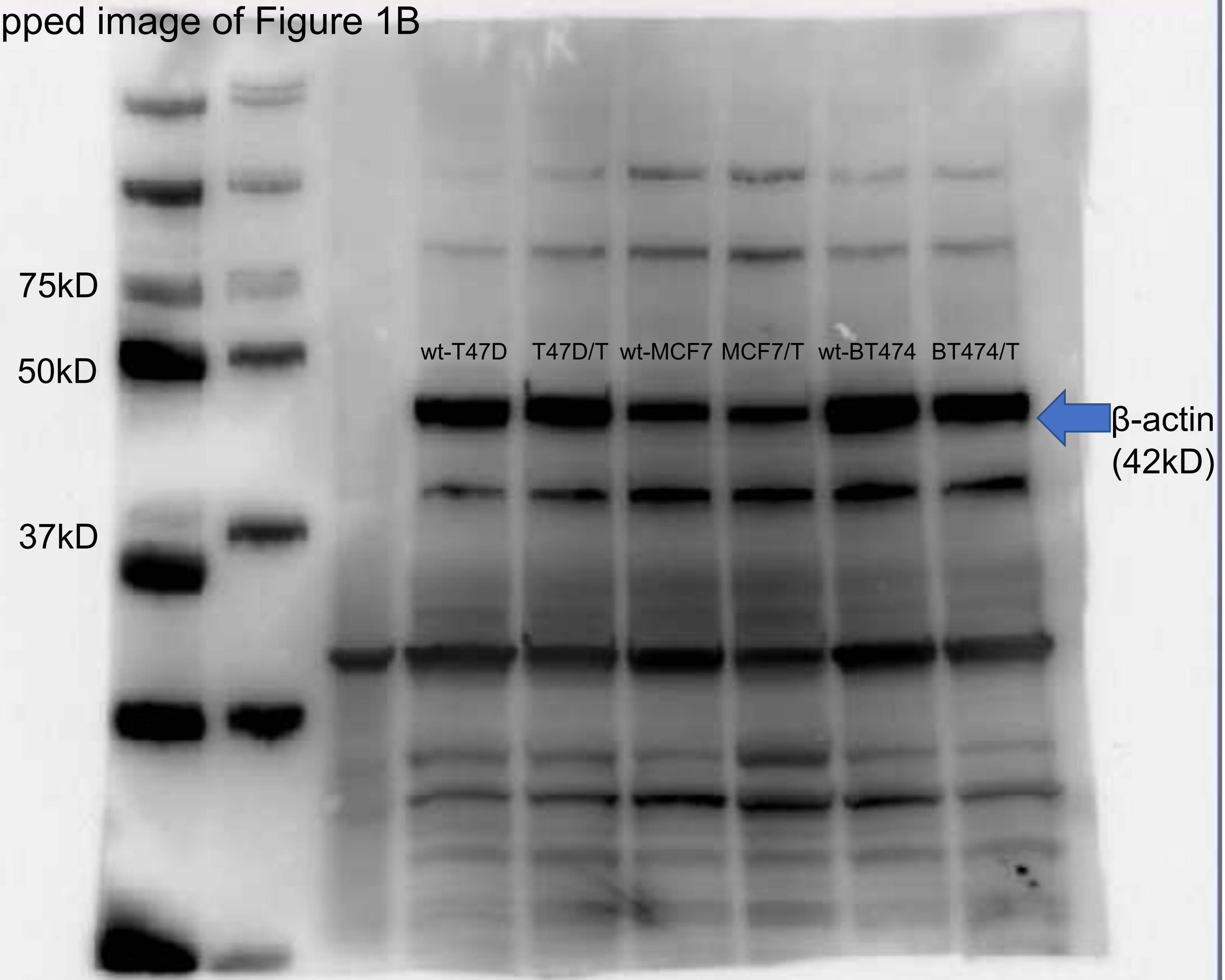

Supplement: S2 File — (PDF) [file pone.0252822.s006.pdf]
